# Impact of the teaching method of the rub-in technique for learning hygienic hand disinfection in medical studies: a comparative effectiveness analysis of two techniques

**DOI:** 10.3205/dgkh000332

**Published:** 2019-11-13

**Authors:** Kenan Dennis Sakmen, Jasmina Sterz, Maria-Christina Stefanescu, Julian Zabel, Marieke Lehmann, Miriam Ruesseler

**Affiliations:** 1 Department of Trauma, Hand and Reconstructive Surgery, University Hospital Frankfurt, Goethe University Frankfurt, Frankfurt, Germany; 2 Department of Pediatric Surgery and Pediatric Urology, University Hospital Frankfurt, Goethe University, Frankfurt, Germany

**Keywords:** hygienic hand disinfection, six-step technique, rub-in technique

## Abstract

**Objective:** Hygienic hand disinfection is of major importance regarding nosocomial infections and antibiotic resistance. The six-step technique is the most commonly taught method, but its superiority has not been empirically demonstrated. This study compares two hand disinfection techniques with regard to their total distribution of the disinfectant.

**Methods:** In this comparative effectiveness analysis, medical students were randomized into two groups. Group 1 was instructed in the 6-step technique, group 2 was referred to a self-responsible application. Learning success was measured using fluorescent disinfectant and black light photographs at three time points (directly, few days later, 5–12 weeks later). Photographs were evaluated quantitatively.

**Results:** 198 students were included in the study (Group 1: 6-step technique; n=103, Group 2: self-responsible disinfection; n=95). 186 were followed up at the second measurement, 182 at the third measurement. Directly after training, there were no significant differences between the two groups. At the second measurement, Group 2 outperformed Group 1 for total, dorsal, and palmar areas (p<0.001, p=0.002, p<0.001). At the third measurement, Group 2 was significantly better (p=0.019) for palmar-sided hands.

In Group 1, areas of disinfected skin deteriorated significantly between measurement 1 and 2 (p=0.019) and measurement 2 and 3 (p<0.001). Group 2 did not deteriorate between measurement 1 and 2 (p=0.269) but between measurement 2 and 3 (p<0.001).

**Conclusions:** Compared to the established six-step technique, a self-responsible application method results in measurably better distribution of the hand disinfectant.

## Zusammenfassung

**Problemstellung:** Die hygienische Händedesinfektion ist in Zeiten von gehäuft auftretenden nosokomialen Infektionen und Antibiotikaresistenzen von größter Bedeutung. Die „6-Schritt-Methode“ ist die verbreitetste Lehrmethode, ihre Überlegenheit ist jedoch nicht evidenzbasiert.

Ziel dieser Arbeit ist, die etablierte 6-Schritt-Methode mit einer eigenverantwortlichen Applikation zu vergleichen.

**Methode:** Im Rahmen der vorliegenden vergleichenden Effektivitätsanalyse wurden Medizinstudierende in zwei Gruppen randomisiert. Gruppe 1 wurde unter Verwendung der 6-Schritt-Methode unterrichtet, während Gruppe 2 zur Desinfektion ohne Vorgabe einer speziellen Reihenfolge oder Methode unterrichtet wurde. Der Lernerfolg wurde an drei Messzeitpunkten (direkt im Anschluss an den Kurs, einige Tage später und nach 5–12 Wochen) unter Verwendung von fluoreszierendem Desinfektionsmittel und Photographien unter Schwarzlicht erhoben.

**Ergebnisse:** Insgesamt wurden 198 Studierenden in die Studie eingeschlossen (Gruppe 1: 6-Schritt-Methode: n=103, Gruppe 2: eigenverantwortliche Desinfektion; n=95). Hiervor nahmen 186 am zweiten Messzeitpunkt und 182 am dritten Messzeitpunkt teil. Direkt nach dem Kurs konnten keine signifikanten Unterschiede zwischen den Gruppen gemessen werden. Am zweiten Messzeitpunkt zeigte Gruppe 2 eine deutlich bessere Verteilung des Desinfektionsmittels, sowohl bezogen auf die gesamte Hand als auch auf die dorsale und palmare Seite (p<0.001, p=0.002, p<0.001). Am dritten Messzeitpunkt zeigte Gruppe 2 eine signifikant bessere Desinfektion auf der palmaren Seite der Hand (p=0.019). Die Studierenden der Gruppe 1 verschlechterten sich signifikant sowohl zwischen dem ersten und zweiten Messzeitpunkt (p=0,019) als auch zwischen dem zweiten und dritten Messzeitpunkt (p<0,001). Die Studierenden der Gruppe 2 zeigten eine konstante Leistung an den ersten beiden Messzeitpunkten (p=0,269), verschlechterten sich lediglich zwischen den Messzeitpunkten zwei und drei (p<0,001).

**Schlussfolgerung:** Die Vermittlung einer eigenverantwortlichen Methode als Lehrmethode für eine hygienische Händedesinfektion resultiert langfristig in einer messbar besseren Verteilung des Desinfektionsmittels als durch die etablierte 6-Schritt-Methode.

## Introduction

Healthcare-associated infections (HCAIs) are a major issue within hospitals [1]. The Point Prevalence Survey initiated by the European Centre for Disease Prevention and Control (ECDC) describes a total prevalence of 6.0% [2]. The World Health Organization (WHO) found that the attributable mortality due to HCAIs in Europe is estimated to be 1% (50,000 deaths per year), but that HCAIs contributes to death in at least 2.7% of cases (135,000 deaths per year) [3]. HCAIs contribute to healthcare system costs due to prolonged hospital stays, increasing resistance of microorganisms to antibiotics, and long-term disabilities [3]. The total annual costs of HCAIs for the health care systems of the EU can be estimated at 7 billion Euro per year, not considering any indirect costs linked to a loss of income as the result of illness and death [4], [5].

Keeping this in mind, it is alarming that one-third of all HCAIs seem to be avoidable [6]. Ninety percent of all HCAIs are transmitted by the hands, thus, hygienic hand disinfection is one of the main preventive actions [6], [7]. Multiple studies have demonstrated that even highly trained healthcare workers are unable to perform adequate hygienic hand disinfection, which is reflected in the finding that cross-infection of patients by trained healthcare workers is the main source of infection [8], [9]. Major factors affecting the compliance and efficacy of hand hygiene include lack of knowledge, institutional guidelines or awareness of guidelines and protocols, experience, and education [3].

Thus, the teaching of techniques for hygienic hand disinfection is of major importance and necessity. Kampf et al. pointed out that different techniques of the rub-in-procedure, especially allowing the individual to use his or her own “responsible application” procedure, lead to a total hand coverage that was at least as good or better than the WHO-recommended six-step technique [3], [10], [11].

Moreover, they could show that, if you compare the recommended six steps to other rub-in techniques, the six steps are insufficient for use in a clinical setting [10]. As a limitation of their study, Kampf et al. acknowledged the rather small group sizes of 15 people recruited in each group. Until now, larger cohort studies are missing. 

The aim of the present study is the comparative effectiveness analysis of two techniques of hand disinfection in undergraduate surgical education. 

## Methods

### Study design

This study has a prospective, comparative effectiveness design with two parallel study arms. We compared the effectiveness of the WHO recommended six-steps technique (DIN EN 1500) and self-responsible application for hygienic hand disinfection.

### Study participants

Participants were voluntary undergraduate medical students at Goethe University in Frankfurt/Main, Germany, in the third year of a 6-year program completing their obligatory surgical training. 

All students were informed of the study content prior to their training. Participation in the study was voluntary and took place after written informed consent had been given, which was revocable at any time. Students were blinded towards the instructional approaches, as well as to the affiliation of their study group. Basic data regarding student age, sex, and duration of medical studies were collected using an online questionnaire.

As stated by the Ethics Board of the Medical Department of Goethe University Frankfurt, Germany, ethical approval was not required for this study. However, the study was conducted in accordance with the Helsinki Declaration.

### Study protocol 

The students were completing their obligatory three-week surgical training, which includes one week training of practical clinical skills at our university and two weeks elective in a surgical ward. The training of practical skills at our skills lab, conducted in small groups of six students per tutor, has been shown to ensure the students have the necessary surgical skills before the start of clinical training and is, therefore, a useful addition to the existing lessons given at hospitals [12]. Prior to their surgical training week, students were allocated to one of the small learning groups by the deanery, independent of the authors and independent of study participation. These learning groups were randomized to one of the two rub-in techniques.

As part of the training week, on their first day, the students must complete a 90-min training course about hygienic behavior in the operating room.

The training starts by refreshing and advancing previous knowledge regarding HCAIs and their prevention, with the main focus on the operating room. Students recapitulated the often insufficiently disinfected areas. Based on the randomization to one of the study groups prior to the training week, as described above, students were then taught based on their study group. The six-step approach group was taught the DIN EN 1500 technique [11], whereas the self-responsible application group was told to disinfect their hands self-responsibly, without being given any particular method of application. Both groups were referred to often insufficiently disinfected areas and were told to take a sufficient amount of the disinfectant (3–5 ml respectively the amount which fits into its own hollow hand). In addition they were taught to respect an application time of at least 30 seconds. The explanation by the peer-lecturer was followed by an exercise phase for each group. 

### Measurement

The first measurement of learning success took place within the teaching unit. The second measurement was done at the end of the one week training of practical clinical skills. Our final measurement was performed at the students’ objective structured clinical examination (OSCE) at the end of the semester. Due to the fact that we had to integrate the study into our curriculum, the third measurement took place 4–12 weeks after the initial training.

At the individual measuring times, each study participant disinfected their hands using a fluorescent disinfectant (10 ml Visirub concentrate + 500 ml Sterillium; Hartmann AG, Germany), which allowed the disinfected areas to be visualized under a black light lamp (Derma LiteCheck Box, Hartmann AG; Germany). Under the black light lamp, photos were taken of the palmar and dorsal sides of each of the student’s hands. For study measurement, it was necessary to process the pictures. Using Adobe Photoshop (San Jose, CA, USA), we blacked out the background and defined the part of the hands, which were relevant for our quantitative analysis. Subsequently, we used the freeware software Image J (Wayne Rasband, NIH, MD, USA) to quantify the non-disinfected portion of the hands. The area could be selected using the colour-threshold function, which enabled us to select the areas by their color, hue, and saturation. We opted to use Image-J because of its usability, financial aspects, and efficacy in the quantitative analysis of this large study population [13], [14].

### Data analysis

The data were evaluated using Microsoft Excel (version 16.9; Microsoft Inc, Redmond, WA, USA), as well as IBM SPSS Statistics 19/22 (SPSS Inc., Chicago, IL, USA) and G * Power [Axel Buchner (University Düsseldorf)]. Because the percent variables to be tested did not have a normal distribution, these data were compared using the nonparametric Kolmogorov-Smirnov test with independent samples. For the tests for significant differences between the three measurement times, the repeated measures parametric analysis of variance (ANOVA) was used, which responded robustly to the violation of the normal distribution assumption. For the tests between the individual times, the Bonferroni correction was applied as a post-hoc procedure. Furthermore, the Cohen’s d effect size was calculated on the basis of the underlying averages and standard deviations for each individual mean or P-value.

## Results

A total of 198 students were included in the study. 186 were followed up at the second measuring point and 182 at the third measuring point (Table 1 (Tab. 1)).

At the first measurement, there were no significant differences in the portions of disinfected skin between the two groups (Table 2). At the second measuring point, Group 2 (self-responsible application) outperformed Group 1 for the total, dorsal, and palmar areas (p<0.001, p=0.002, p<0.001) (Table 2 (Tab. 2)). At the third measurement, Group 2 was significantly better (p=0.019) for the palmar-sided hands (Table 2). The mean scores of disinfected skin areas are given as percentage and standard deviation.

**Figure 1:**
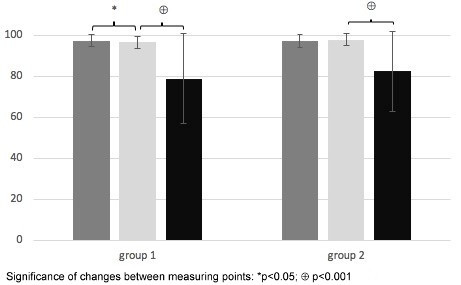
Changes in the areas of disinfected skin areas in time

In Group 1, the areas of disinfected skin deteriorated significantly between measuring point 1 and 2 (p=0.019) a and between measuring point 2 and 3 (p<.001). Group 2 did not deteriorate between measuring point 1 and 2 (p=0.269) but did deteriorate between measuring point 2 and 3 (p<0.001) (Figure 1 (Fig. 1)).

Data are presented as mean scores of disinfected skin areas (percentage and standard deviation). Results at measuring point 1 are presented in dark grey, measuring point 2 in light grey, and measuring point 3 in black.

## Discussion

To decrease HCAIs, every healthcare worker has to be capable of adequate hygienic hand disinfection, which is considered the most effective measure for breaking the chain of infection [15], [16], [17], [18]. Effective hygienic hand disinfection training is essential for improving its practice and should be promoted at all levels of medical training [17], [19].

Despite the major importance of hygienic hand disinfection, few studies analyze the application method and its efficacy or investigate the effect of the teaching method in an educational setting. Medical students have multiple contacts with patients even during the early stages of their undergraduate training, such as bedside teaching, clerkships, and internships. Therefore, an easily recalled method for hand disinfection is highly relevant for the training of future healthcare workers.

The aim of the present study was to identify an easily taught and effectively recalled teaching technique for the hand disinfection for use in undergraduate training. The WHO-recommended six-step technique [3] offers the advantage of algorithm-based learning. An algorithm is defined as a path of action towards the solution of a problem or certain type of problem. During such teaching approaches, a learner is mentally subdividing a competence into several steps in order to memorize it in declarative memory [20].

In comparison to the observation of the peer tutor and application and memorization of a predetermined algorithm, the self-responsible application includes further important steps of the theories of adult learning, helping to anchor the acquired competence even more deeply [21], [22], [23], [24]. Though, the learner is tying the existing knowledge regarding insufficiently disinfected areas with the new learning objective (own hand disinfection) by applying the knowledge directly to the actual-problem-solving process. The learners are actively involved in the learning process through their self-dependent development of an individual algorithm, thereby promoting learning. 

Kampf et al. [10] demonstrated that a self-responsible application is not inferior to other techniques; however, because of the small group sizes in their study-design, they were unable to make significant statements about their findings. On the present study, we were able to confirm the results of Kampf et al. with a much larger number of study participants. Furthermore, we were able to demonstrate the positive effect of the self-responsible technique at the end of the one week training.

In comparison to Kampf et al. [10], a distinctive feature of the present study is that the study was conducted directly within students’ curricular and obligatory training, rather than in an laboratory setting with participants recruited specifically for the purpose of the study.

The greater variation detected at measuring point 3 might be accounted for by the setting of the measuring point: At measuring point 1 and 2, the hand disinfection was the focus of the students’ task; whereas measuring point 3 took place during students’ summative OSCE-setting, where students had to perform various surgical skills, based on which the students are awarded their final grade for surgery. In this setting, the disinfection was not the focus of the activity, but their task in the OSCE setting was to perform an examination of the skull. Thus, it was their own preparation before they start with the surgical examination. However, even though the specific examination setting, 181 from 182 of the students did disinfect their hands even though knowing this was not relevant for passing their OSCE task and achieved 78.93%±22.04% to 82.52%+19.48% of disinfected skin area, which is in this setting a compelling result. Regarding the compliance rates from healthcare workers in the hospital setting, which achieve approximately 70% after training campaigns [25], [26], we hope that the early implementation of hygienic training can help increase the awareness and decrease the number of HCAIs.

We are aware of the limitations of our study. As a single-center study, our findings should be replicated in other settings. The transferability of these findings to a clinical setting was not within the scope of our study and should to be tested in future work. 

## Conclusions

Compared to the established six-step technique, a self-responsible application method results in measurably better long-term disinfection in undergraduate training.

## Notes

### Funding

This work was supported by the German Federal Ministry of Education and Research (grant 01PL12038A) as part of the joint research project “Practical clinical competence – a joint program to improve training in surgery”. 

### Competing interests

The authors declare that they have no competing interests.

## Figures and Tables

**Table 1:**
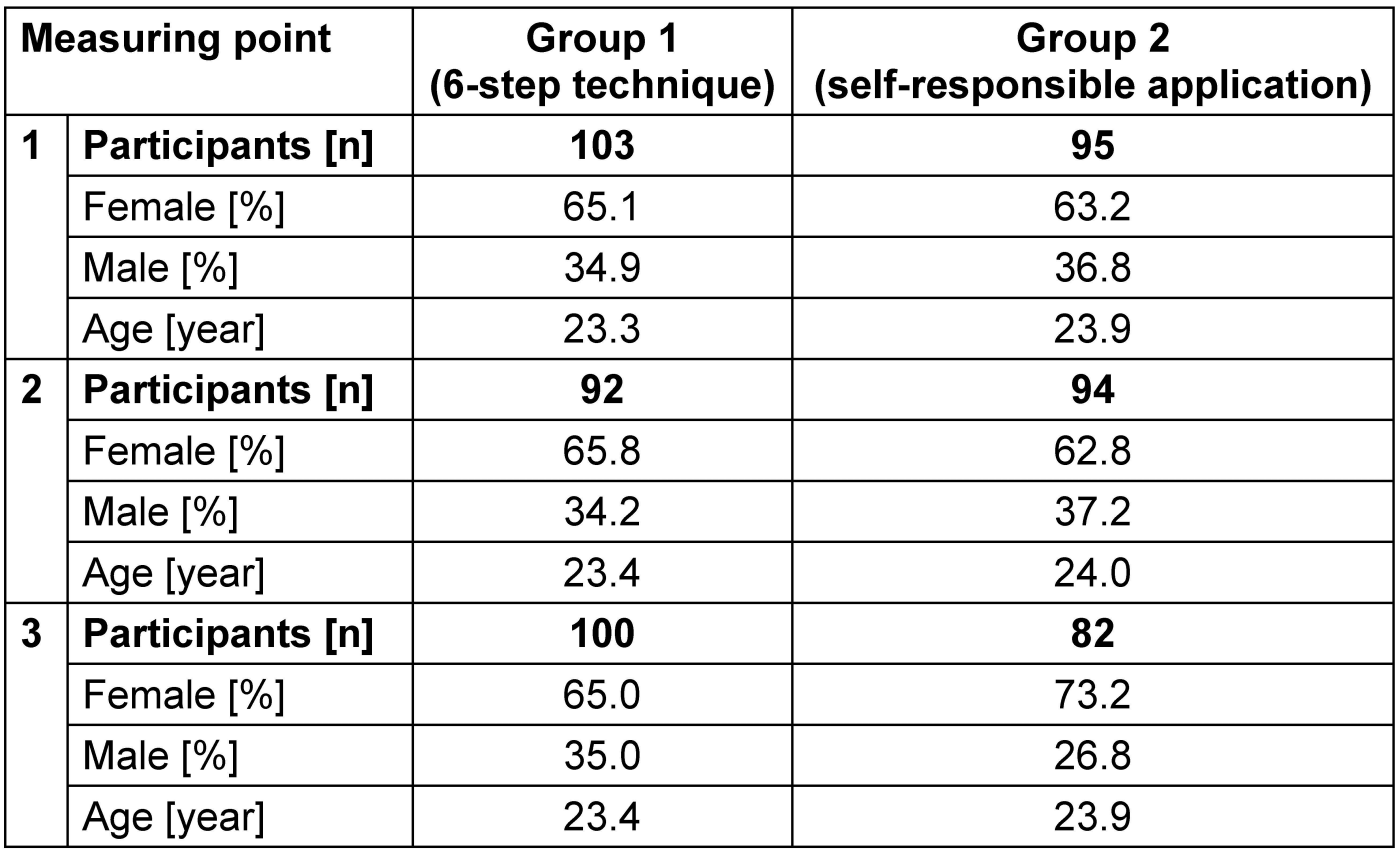
Study participants at the three measuring points

**Table 2:**
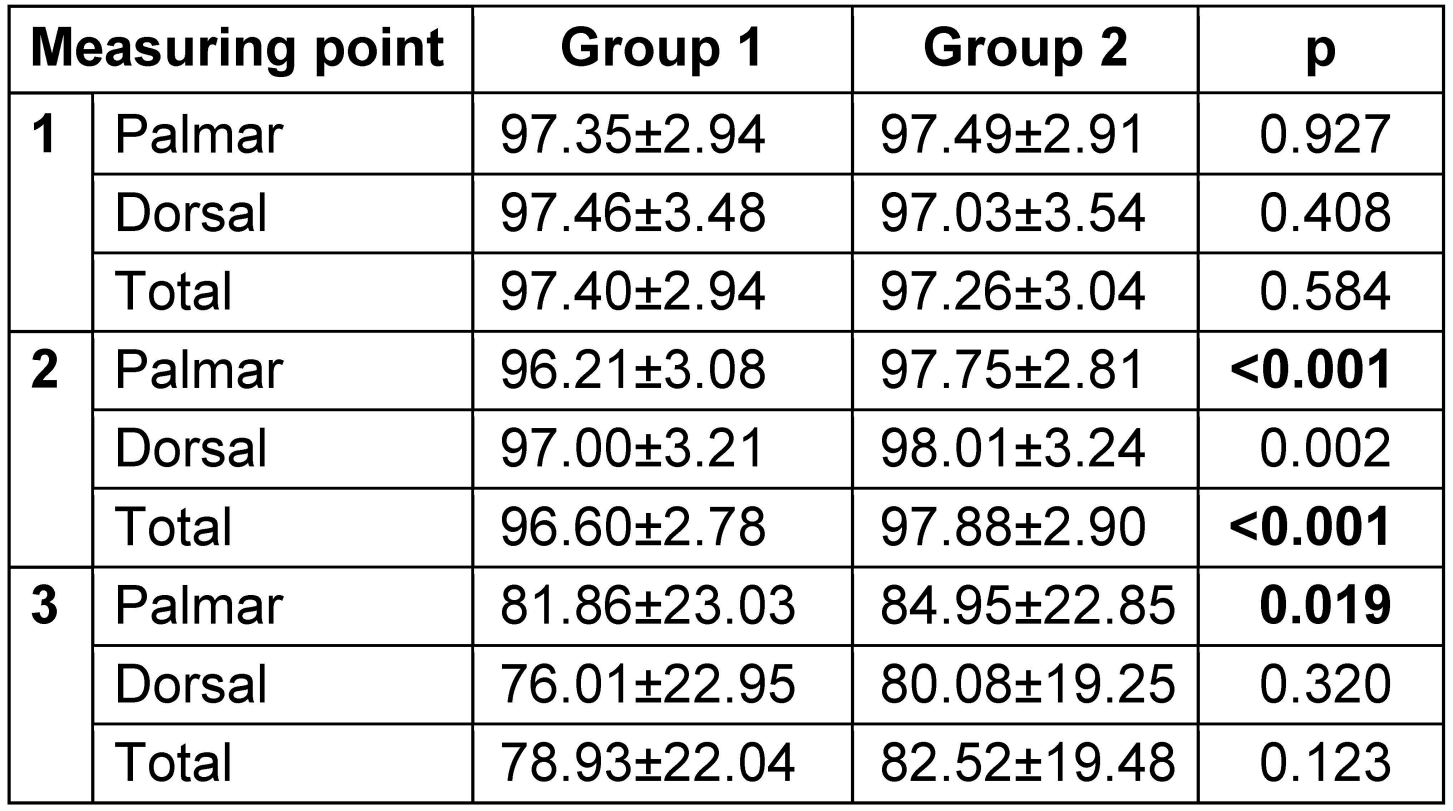
Results of disinfected skin areas by measuring points one, two and three
